# Structural stigma and 7-year improvement in life satisfaction: A repeated cross-sectional study of sexual minority individuals across 28 countries

**DOI:** 10.1093/eurpub/ckac129.196

**Published:** 2022-10-25

**Authors:** R Bränström, J Pachankis

**Affiliations:** 1 Department of Clinical Neuroscience, Karolinska Institutet, Stockholm, Sweden; 2 Department of Social and Behavioral Sciences, Yale School of Public Health, New Haven, USA

## Abstract

**Background:**

Structural stigma toward sexual minority individuals varies widely across countries and is associated with psychosocial health outcomes. Yet, the association of changes in country-level structural stigma over time, as has recently characterized many European countries, with such outcomes is largely unknown. The current study examined the association between change in structural stigma from 2012 to 2019 across European Union countries and change in life satisfaction among sexual minority individuals during the same period. Secondary analyses examined whether changes in structural stigma differentially benefitted some subgroups of sexual minority individuals more than others.

**Methods:**

The current study analyzed data from sexual minority respondents (2012: n = 82,668; 2019: n = 96,576) living in 28 European countries.

**Results:**

Adjusted multilevel models showed that life satisfaction had improved among sexual minority individuals in all countries between 2012 and 2019 (β = 0.32, 95% CI: 0.29, 0.35), but the improvement was stronger among those living in higher stigma countries compared to those living in lower stigma countries. Changes also varied by relationship status; the strongest improvement in life satisfaction as a function of improvement in structural stigma was found among sexual minority individuals in a relationship.

**Conclusions:**

Although life satisfaction has increased during the past decade among sexual minority individuals living in Europe, significant variation in this change exists across countries as a function of country-level structural stigma and individual sociodemographic characteristics. The findings support the relevance of structural stigma for sexual minority individuals’ life satisfaction and call for further research to understand the differential impact of structural stigma across sexual minority subgroups.

